# Reviews

**Published:** 1880-03

**Authors:** 


### Reviews.

---

**A Manual of the Practice of Surgery.** By W. Fairlie Clarke, Assistant Surgeon to Charing Cross Hospital. From the last London edition, Revised and Edited, with additions, by an American Surgeon. New York: William Wood & Co. 1879.

Webster defines a manual as a small book, such as may be carried in "the hand or conveniently handled." In that sense it may be used as a small and convenient book of reference. It does not contain anything in its 300 pages (covering the whole of surgery), that has not been said better and clearer in other books of surgery. Some common prescriptions, and a few recipes for the making of chicken broth and jelly, beef tea, etc., are added which do not make the book much more valuable. M.

---

**Lectures on the Diseases of the Nervous System.** By Professor J. M. Charcot. Translated from the Second Edition by George Sigerson, M. D., M. Ch. Philadelphia: Henry C. Lea.

Any one who has listened to the eminent author of these lectures would readily recognize their general tone, even if the words were ascribed to another person. Charcot's style is as strongly marked in his writings as in his person. There is something rather impressive in the quiet way in which the propositions are stated, and in the judicial fairness with which the conclusions are drawn; while the clearness in presenting the positive knowledge in this obscure department of medicine, is delightfully refreshing.

It would evidently be impossible to condense all the facts relating to the pathology of the nervous system into the two hundred and fifty pages here offered to the student. The general features of the subject, however, are well mapped out, and certain portions, especially the diseases of the spinal cord, are treated in a most exact and original manner. The disorders of nutrition, paralysis, agitans and disseminated sclerosis are considered at length, and five entire lectures devoted to hysteria in its protean forms. These are all clinical lectures in the real sense of the word, and as such contain the very gist of what a practitioner cares most about knowing, H.

---

**Atlas of Human Anatomy.** Illustrating most of the Ordinary Dissections and many not usually practiced by the student, accompanied by an explanatory text. By RICKMAN JOHN GODLEE, Assistant Surgeon to University College Hospital, Senior Demonstrator of Anatomy in University College. Philadelphia: Lindsay & Blakiston.

The third part of this excellent work has made its appearance. It is illustrated, as the two former parts, with four plates, each containing several figures. Plate 1 contains three figures, showing the ordinary dissections to expose the pterygo-maxillary region, and the carotid artery. Plate 2 (10) illustrates in five figures the anatomy of the nose and the neighboring parts. Plate 3 (11) contains in two figures the dissections usually made to expose Mechel's ganglion and the otic ganglion. Plate 4 (12) contains a fine view of the folds of dura mater, with the cranial nerves and intracranial arteries and veins, and several dissections of the orbit. The whole is accompanied with a full explanatory text. The entire work will constitute a perfect and unequalled anatomical atlas, as valuable for the surgeon as for the student.

M.

---

**A Treatise on the Science and Practice of Midwifery.** By W. S PLAYFAIR, M. D., F. R C. P., &c. Third American Edition. With Notes and Additions by ROBERT P. HARRIS, M. D. With two plates and one hundred and eighty-nine illustrations. Philadelphia: Henry C. Lea 1880.

This work of 650 pages, written by a President of the Obstetrical Society of London, is one which bears the stamp of a mind thoroughly acquainted with the subject. It is not a simple compilation, but on the contrary a thorough digest. It is particularly rich in its illustrations, these being both copied and original, and serving greatly to elucidate numerous points. While exceedingly pleased with the whole work, we wish to particularly notice that his views, regarding the frequent use of forceps, correspond with the modern practice, as well as with the conclusions arrived at during a recent discussion upon the subject by the noted obstetricians of Great Britain. It seems now no longer to admit of argument that the forceps in skillful and careful hands is an instrument of which we have long been too fearful. The work is fully up to date and amongst other recent additions contains a chapter upon transfusion. We highly recommend it to students and practitioners. V. P.

---

**Photographic Illustrations of Skin Diseases**. By George Henry Fox, A. M., M. D., Clinical Professor of Dermatology. New York: E. B. Treat, No. 805 Broadway. Parts 5 and 6.

Part 5 contains photographic plates of eczema infantile; eczema papulosum; eczema ichorosum; eczema pustulosum, and eczema squamosum.

Part 6 illustrates eczema barbae; eczema manum; eczema e venis varicosis; ulcus varicosum, and psoriasis annulata.

The author justly says in the preface that "the study of skin diseases without cases, or colored plates, is like the study of osteology without bones, or the study of geography without maps." The profession have so few opportunities to study these diseases in their practice, that plates, prepared with accuracy and skill, constitute an indispensable auxiliary, long needed, and here most successfully accomplished. We can conscientiously recommend these works of Professor Fox. The profession will thoroughly appreciate such helps to the study of a class of disease, otherwise difficult and obscure. L.

**A Treatise on the Theory and Practice of Medicine.** By JOHN SYER BRISTOWE, M. D., F R C. P., Senior Physician, St. Thomas Hospital, etc., etc. Second American Edition Revised by the author, with notes and additions by JAS H. HUTCHINSON, M. D., Physician to the Pennsylvania Hospital, Philadelphia Philadelphia: Henry C. Lea. Cloth, $5.00; Leather, $6.00.

The first edition of this great work of Dr. Bristowe, published in 1877, received the hearty commendation of the American medical Press, and it has been quite extensively adopted as a text book in American Medical Schools. The new edition is a portly volume of nearly 1100 closely printed pages. A chapter on insanity has been added, and the author has made such corrections and additions to the body of the work as seemed advisable in the light of the latest experience. The reader will find every conceivable subject connected with the practice of medicine, ably presented in a style at once clear, interesting and concise. The additions made by Dr. Hutchinson are appropriate and practical, and greatly add to its usefulness to American readers. D.

---

**A Clinical Treatise on Diseases of the Liver.** By Dr. FRIED. THEOD. FRERICHS. In three volumes. Vol. I. Translated by Charles Murchison. M. D., F. R. C. P. New York: Wm. Wood & Co., 27 Great Jones street, 1879.

The first chapter on the *Historical Account* of disease of the liver is full of instruction and interest, suggestive of the great change of views which have taken place as to the nature of most all diseases. This first chapter gives the history of the physiology of the liver, and of the pathology of this organ with the present condition of our knowledge, and new questions for investigations, with most important literature on the subject, and is so full of suggestions and instruction as to commend the work to the careful attention of the members of the profession, old and young; old, because of the crude and untenable opinions they have entertained; young, because of the present views, now so fully demonstrated to be correct. All the other chapters are of equal interest. J. F. M.

